# Quantification of CH-π Interactions Using Calix[4]pyrrole Receptors as Model Systems

**DOI:** 10.3390/molecules200916672

**Published:** 2015-09-14

**Authors:** Gemma Aragay, Daniel Hernández, Begoña Verdejo, Eduardo C. Escudero-Adán, Marta Martínez, Pablo Ballester

**Affiliations:** 1 Institute of Chemical Research of Catalonia (ICIQ), Avgda. Països Catalans 16, Tarragona 43007, Spain; E-Mails: garagay@iciq.es (G.A.); dhernandez@iciq.es (D.H.); begona.verdejo@uv.es (B.V.); eescudero@iciq.es (E.C.E.-A.); mmartinez@iciq.es (M.M.); 2 Catalan Institution for Research and Advanced Studies (ICREA), Passeig Lluís Companys, 23, Barcelona 08018, Spain

**Keywords:** CH-π interactions, calix[4]pyrrole, trimethylamine oxide, trimethylphosphine oxide, electrostatic forces, dispersion forces

## Abstract

We describe the use of two series of aryl-extended calix[4]pyrrole receptors bearing two and four electronically tunable phenyl groups, respectively, in their *meso*-positions as model systems for the quantification of CH-π interactions in solution. The “four-wall” and the “two-wall” receptors formed thermodynamically stable 1:1 complexes in acetonitrile solution with both trimethylamine *N*-oxide and trimethylphosphine *P*-oxide as guests. The complexes were mainly stabilized by the formation of four convergent hydrogen bonds between the oxygen atom of the guests and the pyrrole NHs of the host. In general, the *N*-oxide produced thermodynamically more stable hydrogen bonding interactions than the *P*-oxide. Upon guest binding, the receptors adopted the cone conformation and the methyl groups of the included guests engaged in CH-π interactions with the aromatic walls. We show that the modification of the electronic properties of the aromatic surfaces, in any of the receptor series, did not have a significant impact in the measured binding affinities for a given guest. However, the larger binding affinities determined for the “four-wall” receptors in comparison to the “two-wall” counterparts supported the importance of CH-π interactions on guest complexation. The strength of the CH-π interactions present in the inclusion complexes was quantified employing the octamethyl calix[4]pyrrole as reference. We determined an average magnitude of ~1 kcal·mol^−1^ for each CH-π interaction. The CH-π interactions featured a reduced electrostatic nature and thus dispersion forces were assigned as main contributors of their strength.

## 1. Introduction

Some authors have considered CH-π interactions as weak or “non-conventional” hydrogen bonding interactions wherein a CH group acts as the hydrogen bond donor and the electrons of a π-system as acceptors [1]. However, the nature of hydrogen bonding interactions and CH-π interactions seems to be remarkably different. Whereas conventional hydrogen bonds are mainly dominated by electrostatic forces, “typical” CH-π interactions are thought to have a major contribution from dispersion forces being their electrostatic component almost insignificant [2]. It is worth noting that CH-π interactions involving “activated” CH bonds, that is having highly acidic protons (e.g., CHCl_3_ and acetylene), may possess more similarities to conventional hydrogen bonds in terms of (a) the importance of the electrostatic contribution and (b) the directionality of the interaction. Nevertheless, the magnitude of any of the two types of CH-π interactions, typical or activated, is considerably smaller than that of a conventional H-bond. Thus, an isolated and conventional neutral H-bond in chloroform solution produces an enthalpy gain that ranges from 1 to 7 kcal·mol^−1^. Conversely, the enthalpy of a single CH-π interaction is supposed to be less than 1 kcal·mol^−1^ [1,3]. In spite of the low enthalpic contribution and in close analogy with other intermolecular interactions, the total energy provided by CH-π interactions is significantly increased by the participation of multiple CH and/or π-groups acting in concert in the final assembly (multivalency). The analogy that exists between “typical” CH-π interactions and van der Waals forces suggests that the role of the former in controlling the relative orientation of molecules in a supramolecular complex or even molecular conformations could be insignificant. However, and as stated above, the occurrence of multiple CH-π interactions in a supramolecular assembly renders them quite important in terms of total energy and preferred structure. Consequently, CH-π interactions have been described to play important roles in a variety of phenomena including molecular recognition, crystal engineering and the structure of many biomolecules [4,5,6,7].

The initial findings suggesting the existence of these weak interactions were put forward by Andreetti and coworkers in 1979. They reported the crystal structure of a calix[4]arene receptor that included a toluene molecule inside its cavity [8]. The authors attributed the stability of the complex to the multiple weak CH-π interactions established between the methyl groups of the *t*-butyls located at the upper rim of the calix[4]arene and the aromatic ring of the toluene molecule included in the cavity. Additional CH-π interactions were also present between the methyl group of the toluene guest and the aromatic panels of the calix[4]arene host.

Since then, many experimental, computational and solid-state studies supporting the existence of attractive CH-π interactions have been published. In 1999, Umezawa and collaborators reported, following an accurate statistical analysis of the Cambridge Crystal Structure Database (CCSD), that a large number of organic crystals displayed short contacts between CH bonds and π-systems (shorter distance than their Van der Waal radii) [9]. However, the nature and physical origin of the attraction between a CH bond and a π-system, as well as the role that it plays in chemical and biological systems are not totally established. A better understanding of the nature of the CH-π interactions, both from a theoretical or experimental point of view, will certainly benefit our knowledge in the design and development of new functional supramolecular systems and materials and further our skills in the field of drug design.

The “molecular torsion balance” approach originally developed by Wilcox [10], and latter applied by Diederich [11] constituted a useful physical organic tool for the evaluation of the magnitudes and forces involved in CH-π interactions. Surprisingly, quite different conclusions regarding the importance of electrostatic forces in CH-π interactions were drawn from the studies mentioned above. On the one hand, Wilcox concluded that in chloroform solution the electronic nature of the *para*-substituent (electron withdrawing or electron donating) of the aromatic rings (π-system) and consequently their electrostatic potential had a reduced effect on the strength of the CH-π interaction and thus electrostatics was not a dominant force of the interaction [12]. On the other hand, working in benzene solution, Diederich found a linear correlation with a steep slope between the free energies values measured for CH-π interactions and the electronic properties of the aromatic ring (π-system) defined by the electronic nature of the *meta* substituents [11]. Hunter and Cockroft [13] reasoned that the observed differences in the two studies could be reconciled considering desolvation effects (*i.e.*, benzene and chloroform). In this particular case, the take home message was that the solvation of the π-surface by a single molecule of solvent featuring superior hydrogen bond donor properties than the two CHs involved in the CH-π interactions faded the electrostatic component of the latter.

More recently, other authors have tried to dissect the contribution of the dispersive and electrostatic components to the overall stability provided by CH-π interactions. They employed a strategy based on dynamic combinatorial chemistry [14,15]. They concluded that the electrostatic contribution of the CH-π interactions in water could be modulated by increasing the acidity of the CH groups.

Recently, we became interested in the experimental quantification of anion-π interactions [16,17,18]. We used “two-wall” and “four-wall” aryl extended calix[4]pyrrole receptors as model systems to investigate the magnitude of the anion-π interactions. The receptors used featured phenyl aromatic walls with different electronic properties [16]. We obtained a nice linear relationship between the free energies estimated for the anion-π interactions and the electrostatic surface potential (ESP) value of the aromatic ring. This observation indicated that anion-π interactions were mainly dominated by electrostatics.

**Figure 1:**
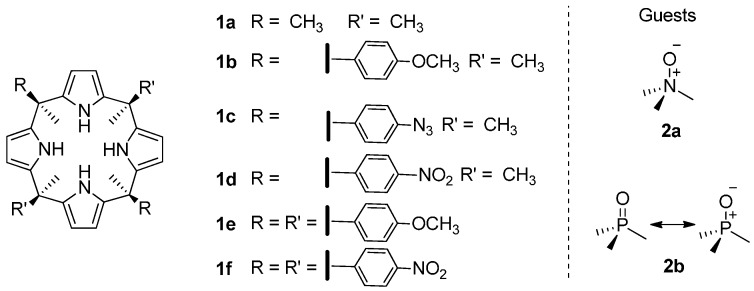
Molecular structures of the calix[4]pyrrole receptors (**1a**–**1f**) and guests (**2a** and **2b**) used in this work.

In a closely related manner, herein we describe our efforts to unravel the physical nature and the strength of the CH-π interactions occurring in the inclusion complexes of a series of aryl extended calix[4]pyrrole receptors with trimethylamine *N*-oxide **2a** and trimethylphosphine *P*-oxide **2b**. We selected three “two-wall” and two “four-wall” calix[4]pyrrole receptors to undertake the study. The selected receptors bear a single electron-withdrawing or electron-donating group in the *para*-position of their *meso*-phenyl substituents. The different electronic characteristics of the *para*-substituent are used to tune the electrostatic potential of the aromatic surface (Figure 1). The two guests, **2a** and **2b**, used in the study are known to establish four convergent hydrogen bonds with the NHs of the calix[4]pyrrole core, both in polar and non-polar solvents. This host–guest interaction locks the receptor in the cone conformation [19]. The methyl groups of the bound guest are adequately oriented to engage in CH-π interactions with the aromatic walls of the host. We demonstrate that the existence of multiple CH-π interactions is responsible for the high thermodynamic stabilities determined for the inclusion complexes. The small magnitude of the slope (close to zero) for the linear correlation of the experimentally determined free energy values for the CH-π interactions (ΔΔ*G*) *vs.* the ESP values at the center of the phenyl rings indicated that electrostatic factors are not determinant in the interaction strength. Most probably, the low hydrogen bond donor abilities of both guests, which qualify the interactions as “typical” CH-π interactions (“non-activated”), are responsible for the poor contribution of the electrostatic forces. We assign the nature of the CH-π interactions present in the studied model systems mainly to polarizability effects and London dispersion forces.

## 2. Results and Discussion

### 2.1. Binding of Octamethyl Calix[4]pyrrole ***1a*** with Guests ***2a*** and ***2b***. Definition of the Reference System

Hunter suggested the use of Equation (1) to predict free energy values for single hydrogen bonding interactions (ΔΔ*G_HB_* in kcal·mol^−1^) in any solvent [20].

ΔΔ*G_HB_ =* [−(α − α*_s_*)(β − β*_s_*) + 6]/4.18 (1)

**Figure 2:**
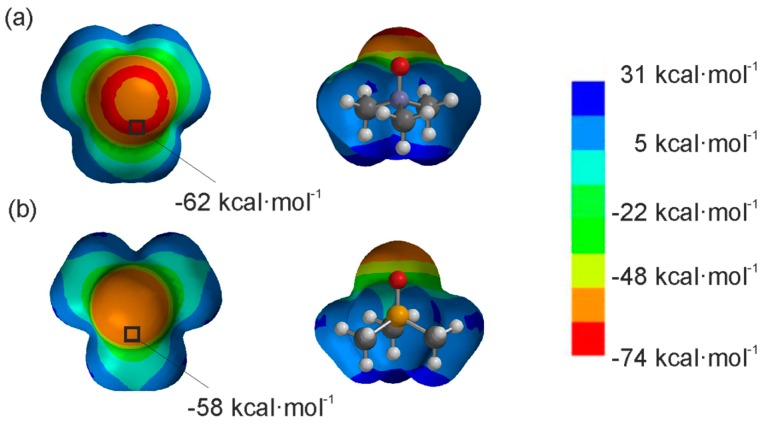
Top and side views of the representation of the electrostatic potential on the van der Waals surface for trimethylamine oxide **2a** (**a**) and trimethylphosphine oxide **2b** (**b**). The surface was calculated by using B3LYP/6-31G* in vacuum [24]. The location and the value for the minimum ESP are specified in each case.

α and β are experimentally determined descriptors used to quantify the hydrogen-bonding donor and acceptor properties, respectively, of functional groups and molecules. In Equation (1), α_s_ and β_s_ refer to the corresponding values of the solvent. Studies performed by Hunter *et al.*, demonstrated the existence of a linear relationship between α and β values and the magnitudes of the maxima and minima, respectively, calculated on the molecular electrostatic potential surfaces of molecules at the AM1 level of theory [21,22]. The relevant α and β values of pyrrole, *N*-oxide **2a**, *P*-oxide **2b** and acetonitrile are: α(pyrrole) = 3.0, α_s_(CH_3_CN) = 1.7, β_s_(CH_3_CN) = 5.1, α(**2a**) = 1.2 β(**2a**) = 12.2, α(**2b**) = 1.1, β(**2b**) = 10.7. These data indicate that *N*-oxide derivatives are strong neutral hydrogen-bond acceptors. The substitution of the nitrogen atom by a phosphorous atom generates analogous phosphine oxide derivatives, which are weaker hydrogen bond acceptors. Considering the formation of a single hydrogen bond interaction between pyrrole and *N*-oxide **2a**, the estimated free energy (ΔΔ*G_HB_*) of the interaction in acetonitrile solution is approximately −0.77 kcal·mol^−1^ (K = 3.7 M^−1^). Changing the hydrogen-bond acceptor partner to the *P*-oxide **2b**, the free energy for the formed hydrogen bond drops to ΔΔ*G_HB_* = −0.31 kcal·mol^−1^ (K = 1.7 M^−1^). Although Equation (1) is not suitable for the prediction of free energies of complexes stabilized by hydrogen bonding arrays, we anticipated from the results above that calix[4]pyrrole receptors would bind *N*-oxide **2a** stronger than *P*-oxide **2b**. Both guests are expected to form four convergent hydrogen bonds between their oxygen atoms and the pyrrole NHs of the receptor in cone conformation. Figure 2 depicts the electrostatic potential surfaces for **2a** and **2b** calculated at the B3LYP/6-31G* level of theory [23]. A more negative ESP value is observed on the oxygen atom of the trimethylamine oxide **2a** compared to the one in the trimethylphosphine oxide **2b**. This result is in agreement with the previously described α and β values based on AM1 calculations.

The theoretical predictions described above were confirmed by experiment. We used ^1^H-NMR (proton nuclear magnetic resonance) spectroscopy to probe the binding of octamethyl calix[4]pyrrole **1a** with trimethylamine oxide **2a** and trimethylphosphine oxide **2b** in CD_3_CN solution.

Incremental additions of trimethylamine oxide **2a** to a millimolar solution of octamethyl calix[4]pyrrole **1a** in CD_3_CN produced reduced changes in the proton signals of free host and guest (Figure S1). The most significant chemical changes were observed on the pyrrole NHs resonating at 7.4 ppm in free **1a**. The pyrrole NH experienced a downfield shift upon addition of **2a** (Δδ ~ 2 ppm). The gradual change of the NH chemical shift indicated that the chemical exchange between free and bound **1a** occurred at a rate that was fast on the ^1^H-NMR timescale. In addition, the downfield shift experienced by the NHs was consistent with establishment of hydrogen bond interactions between the four pyrrolic NHs and the oxygen atom of the *N*-oxide **2a**. A less pronounced downfield shift of the pyrrole NHs was detected when trimethylphosphine oxide **2b** was employed as guest in a ^1^H-NMR titration of **1a** (Figure S3). The quantitative assessment of the binding affinities of the formed complexes was performed by fitting the ^1^H-NMR titration data to a simple 1:1 binding model using the HypNMR2008 software [25] (Figures S2 and S4). In both cases, we obtained a good fit and the calculated binding constant values were K(**2a@1a**) = 72.2 ± 14.0 M^−1^ and K(**2b@1a**) = 8.9 ± 1.8 M^−1^.

The free energies of binding determined for the **2a@1a** and **2b@1a** complexes (Δ*G* = −2.5 ± 0.1 and −1.3 ± 0.1 kcal·mol^−1^) are suitable estimates of the hydrogen bonding interactions responsible of the stabilization of the complexes. These values will be used as reference for dissecting the contribution of CH-π interactions in the thermodynamic stability of the inclusion complexes of guest **2a** and **2b** with aryl extended calix[4]pyrroles (*vide infra*).

### 2.2. Binding of “Two-Wall” Calix[4]pyrroles with Guests ***2a*** and ***2b***

**Figure 3:**
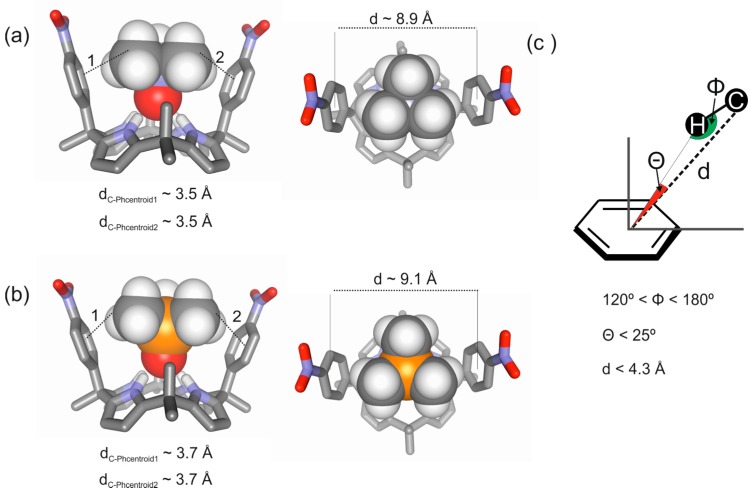
Energy minimized structures for the inclusion complexes of a “two-wall” aryl-extended calix[4]pyrrole with trimethylamine *N*-oxide **2a** (**a**) and with trimethylphosphine *P*-oxide **2b** (**b**) obtained at the BP86/def2-SVP level of theory using TURBOMOLE version 7.0 software [26]; (**c**) Schematic representation of the CH-π interactions occurring in the complexes highlighting three geometric parameters (d, Φ, Θ) typically used to characterize them in previously reported computational studies [2].

The α,α-isomers of “two-wall” aryl extended calix[4]pyrrole receptors adopt the cone conformation upon binding guests having electron rich atoms, such as the oxygen atom of the trimethylamine oxide **2a**. In this conformation, the *meso*-phenyl substituents of the host and the methyl groups of **2a** are properly oriented and at distances suitable to establish CH-π interactions. The binding of trimethylphosphine oxide **2b** with “two-wall” receptors follows an analogous behavior (Figure 3).

**Figure 4:**
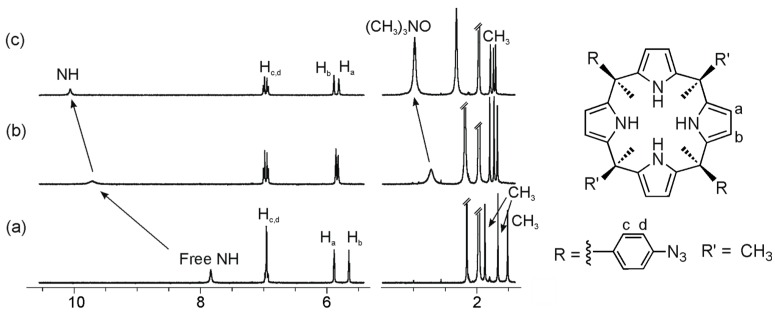
Selected regions of the ^1^H-NMR (Proton Nuclear Magnetic Resonance) spectra of a 1.6 mM CD_3_CN solution of calix[4]pyrrole **1c** with 0 eq (**a**); 2 eq (**b**); and 8 eq (**c**) of **2a** added.

The ^1^H-NMR spectra of “two-wall” calix[4]pyrroles, **1b**–**1d**, in CD_3_CN solution, showed two sharp and well-resolved signals for the β-pyrrolic protons (H_a_ and H_b_) resonating at δ ~ 5.8 ppm. The pyrrolic NHs appeared as a broad band at δ ~ 8 ppm. This signal was significantly downfield shifted with respect to chemical shift value determined for the pyrrole NHs of the same receptors in CDCl_3_ solution. Finally, three sharp singlets could be observed in the upfield region of the spectra (between δ ~ 1.5 and 2 ppm) that corresponded to three pairs of chemically non-equivalent *meso* methyl protons integrating for a 1:1:1 ratio (Figure 4a). The number of proton signals was in agreement with a *C*_2v_ symmetry for the host. However, the NMR information was not useful to conclude if the receptors were locked in alternate or vase conformation or they were experiencing an interconversion between conformers that was fast on the NMR timescale. Based on the solid-state structure of these compounds [17], we inferred that in acetonitrile solution the pyrrole NHs were hydrogen bonded to two solvent molecules and the receptor was in the 1,3-alternate conformation.

The incremental addition of trimethylamine oxide **2a** to a millimolar solution of the “two-wall” calix[4]pyrrole **1b(**R = OMe) in CD_3_CN provoked chemical shift changes in most of its proton signals (Figure S5). The pyrrole NH protons experienced a gradual and large downfield shift (Δδ > 2 ppm). This observation suggested their involvement in four convergent hydrogen bonding interactions with the oxygen atom of bound **2a**. In agreement with this binding geometry, the signal of the methyl protons of the bound guest resonated upfield (Δδ ~ −0.5 ppm) in comparison with that of the free guest. The methyl protons of bound **2a** moved upfield owing to the magnetic shielding exerted by the aromatic panels of the host in the formed inclusion complex.

The observation of a single set of proton signals for the calix[4]pyrrole receptor **1b** throughout the titration indicated that the chemical exchange between free and bound host was fast on the ^1^H-NMR chemical shift timescale. It was necessary to add more than 10 equiv of **2a** to reach binding saturation. The ^1^H-NMR titration data were analyzed using the HypMNR2008 [25] software and a simple theoretical 1:1 binding model. The fit of the theoretical curve to the experimental data was good and returned an association constant value of *K***_2a@1b_** = 5.2 ± 1.3 × 10^3^ M^−1^ for the **2a@1b** complex (Figure S6).

Interestingly, the association constant value determined for the **2a@1b** complex was two orders of magnitude larger than the one measured for the same guest **2a** binding with octamethyl calix[4]pyrrole **1a** (*vide supra*). This increase on binding affinity displayed by the “two-wall” receptors was attributed to attractive CH-π interactions occurring between the methyl groups of the included guest and the electron-rich *meso*-phenyl groups of **1b**.

We also assessed the binding constant values of the inclusion complexes of **2a** with other “two-wall” calix[4]pyrrole receptors having aromatic walls with different electronic properties (Figure 4 and Table 1) [27,28]. The substitution of the electron-donating *para*-OMe groups in the *meso*-aryl rings by electron-withdrawing –N_3_ (**1c**) or –NO_2_ groups (**1d**) produced a significant reduction of the negative electrostatic surface potential (ESP) value at the center of the phenyl ring (Figure S17) [24]. It is well established that intermolecular interactions with aromatic systems that are mainly based on electrostatic forces display linear free energy (Δ*G*) relationships with respect to the Hammett constants of the aromatic substituents (Hammett plots). Typically, σ_p_ values are used in these free energy plots (Hammett plots). We as well as others have shown that σ_p_ and σ_m_ values correlated linearly with the ESP values at the center of the aromatic rings. In this study, we will correlate ΔΔ*G* values *vs.* ESP values at the center of aromatic rings to construct pseudo Hammett plots.

Analogous binding studies were also performed with calix[4]pyrroles **1b**–**d**. The three aryl extended calix[4]pyrrole receptors **1b**–**d** were also titrated against trimethylphosphine oxide **2b** (Figures S5–S16). The determined binding constants for all the investigated complexes are summarized in Table 1.

**Table 1:** Electrostatic Surface Potential (ESP) values (kcal·mol^−1^), association constant values (K_a_, M^−1^), free energies of complexation (Δ*G*, kcal·mol^−1^) measured in CH_3_CN at 298 K for the 1:1 complexes of guests **2a** and **2b** with calix[4]pyrroles **1a**–**f** and free energy values calculated for CH-π interactions (ΔΔ*G*, kcal·mol^−1^ = Δ*G*(**2a**–**b**@**1b**–**d**) − Δ*G*(**2a**–**b**@**1a**).

	ESP ^a^	2a			2b		
		K_a_	−Δ*G*	−ΔΔ*G*	K_a_	−Δ*G*	−ΔΔ*G*
**1a**	-	7.22 ± 1.4 × 10^1 b^	2.53	-	8.91 ± 1.8	1.30	-
**1b**	−20.7	5.15 ± 1.3 × 10^3 b^	5.06	2.51 ± 0.19	8.21 ± 0.9 × 10^1 b^	2.61	1.32 ± 0.13
**1c**	−12.9	3.87 ± 0.4 × 10^3 b^	4.89	2.36 ± 0.12	7.00 ± 0.8 × 10^1 b^	2.52	1.22 ± 0.14
**1d**	−2.0	4.72 ± 0.9 × 10^3 b^	5.01	2.52 ± 0.16	7.98 ± 1.6 × 10^1 b^	2.59	1.30 ± 0.17
**1e**	−20.7	1.41 ± 0.1 × 10^5 c^	7.02	4.49 ± 0.12	9.60 ± 0.9 × 10^1 b^	2.70	1.41 ± 0.13
**1f**	−2.0	1.06 ± 0.1 × 10^5 c^	6.85	4.32 ± 0.12	2.57 ± 0.3 × 10^2 b^	3.29	2.00 ± 0.14

^a^: Value of the electrostatic surface potential at the center of the aromatic ring calculated in vacuum;

^b^: Determined by ^1^H-NMR spectroscopic titration; ^c^: Determined by ITC.

Several conclusions can be drawn from the four first entries presented in Table 1: (a) “two-wall” aryl extended calix[4]pyrroles form inclusion complexes with guests **2a** and **2b** that are two and one order of magnitude more stable, respectively, than their complexes with octamethyl calix[4]pyrrole **1a**; (b) the magnitude of the binding constants of aryl extended calix[4]pyrroles does not change significantly with the modulation of the electronic properties of the aromatic ring; and (c) the complexes with the *N*-oxide **2a** are thermodynamically more stable than those with the *P*-oxide **2b**.

Taken together, our data indicated that *N*-oxide **2a** formed stronger hydrogen bonding interactions than the *P*-oxide **2b**. This result is in complete agreement with the superior hydrogen bonding acceptor properties of *N*-oxide **2a** (β = 12.2) with respect to the *P*-oxide **2b** (β = 10.7). We also observed that the contribution of the hydrogen bonding and CH-π interactions to the total free energy of the aryl extended calix[4]pyrrole complexes is equally balanced for both guests. However, the hydrogen bonding and CH-π interactions for the *N*-oxide **2a** are stronger than for the *P*-oxide **2b**. It is worth noting here that our results demonstrate that multiple CH-π interactions contribute energetically equally than four hydrogen bonding interactions in the stabilization of the complex. Because the hydrogen donor properties of **2a** (α = 1.2) and **2b** (α = 1.1) are similar, we assigned the measured difference in free energies for their CH-π interactions to changes in the geometry of the interaction caused by the larger size of the *P*-oxide and differences in the residual vibrational motions of the complexes deriving from dissimilar thermodynamic stabilities. The difference in the strength of the hydrogen bonding interactions of the two guests was explained in the previous section.

**Figure 5:**
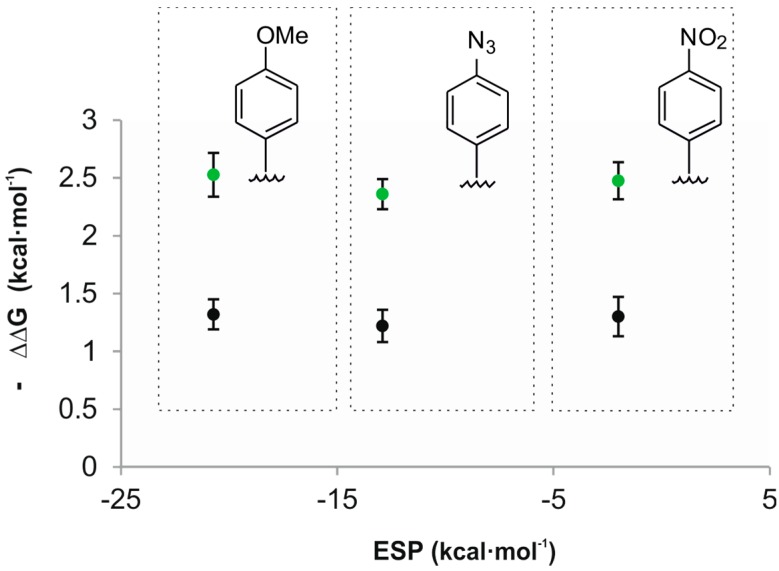
Plot of the experimental free energy values calculated for the CH-π interactions of guests **2a** (**black**) and **2b** (**green**) *vs.* the ESP values calculated in vacuum at the centroid of the aromatic rings using a model system based on “two-wall” calix[4]pyrrole receptors.

Acetonitrile is better hydrogen bond donor (α = 1.7) than the studied oxide guests and is expected to compete with them for the CH-π interactions. Consequently, theory predicts that the plot of the free energy values of the CH-π interactions *vs.* ESP values of the aromatic rings should have a significant slope [29]. Experimentally, we determined that the plot of the calculated free energies for the CH-π interactions *vs.* ESP values of the aromatic rings had a slope close to zero (Figure 5). A likely explanation of this observation is that we are dealing with “typical” CH-π interactions owing to the low alpha values of both guests. The nature of “typical” CH-π interactions is not based on electrostatic forces but mainly on London dispersion forces.

### 2.3. Binding Studies Using “Four-Wall” Calix[4]pyrrole Receptors and Guests **2a** and **2b**

The introduction of two additional *meso*-aromatic walls in the calix[4]pyrrole receptors is expected to increase the number of possible CH-π interactions in the complexes, and consequently it should translate in a larger thermodynamic stability of the resulting complexes.

**Figure 6:**
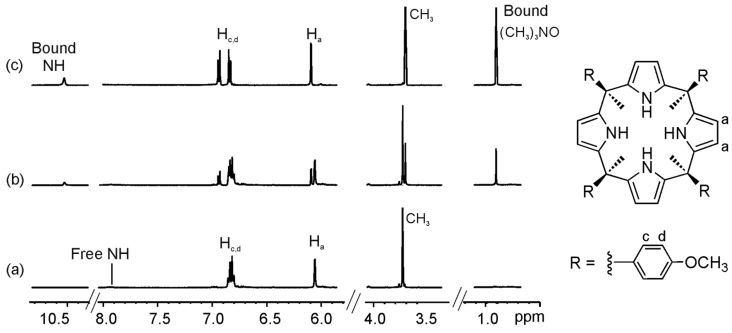
Selected regions of the ^1^H-NMR spectra of a CD_3_CN solution of calix[4]pyrrole **1e** with 0 eq (**a**); 0.4 eq (**b**); and 1 eq (**c**) of **2a** added.

We selected two “four-wall” calix[4]pyrrole receptors **1e**–**f** that are analogous to the “two-wall” calix[4]pyrroles **1b** and **1d** with –OMe and a –NO_2_ groups in the *para* position of their *meso*-aryl rings. Addition of 0.4 equiv of guest **2a** to a millimolar solution of calix[4]pyrrole **1e** in CD_3_CN solution, provoked the emergence of a new set of proton signals in the ^1^H-NMR spectrum that was attributed to the bound host in the inclusion complex **2a**@**1e** (Figure 6 and Figure S18). Additionally, we observed a new and sharp singlet resonating at δ = 0.9 ppm that corresponded to the included guest. Addition of 1 equiv of **2a** resulted in the observation of only the set of proton signals corresponding to the bound host and guest in the **2a**@**1e** complex. The addition of more than 1 equiv of **2a** did not induce additional changes to the proton signals of the complex but a new signal at 3.1 ppm emerged, which corresponded to the free guest **2a**. Altogether, these observations indicated that the equilibrium between bound and free **1e** was slow on the ^1^H-NMR timescale and that the association constant for the **2a**@**1e** complex in CD_3_CN was higher than 10^4^ M^−1^. Similar results were obtained for the binding studies of receptor **1f** with **2a** (Figure S22).

For the accurate determination of the binding constant values of the complexes of the “four-wall” receptors and **2a**, we performed isothermal titration calorimetry (ITC) experiments. The ITC experiments showed excellent fits to the theoretical binding isotherm for the formation of a 1:1 complex (Figures S19 and S23). The returned association constants (Table 1) were approximately two orders of magnitude higher than the ones obtained with the “two-wall” counterparts.

The ΔΔ*G* values calculated for the CH-π interactions in the **2a@1e** and **2a@1f** complexes were almost double than the one calculated for the “two-wall” counterparts. This result was in agreement with the increase on the number of π systems (aromatic walls) displayed by **1e**–**f** with respect to **1b**–**d**.

**Figure 7:**
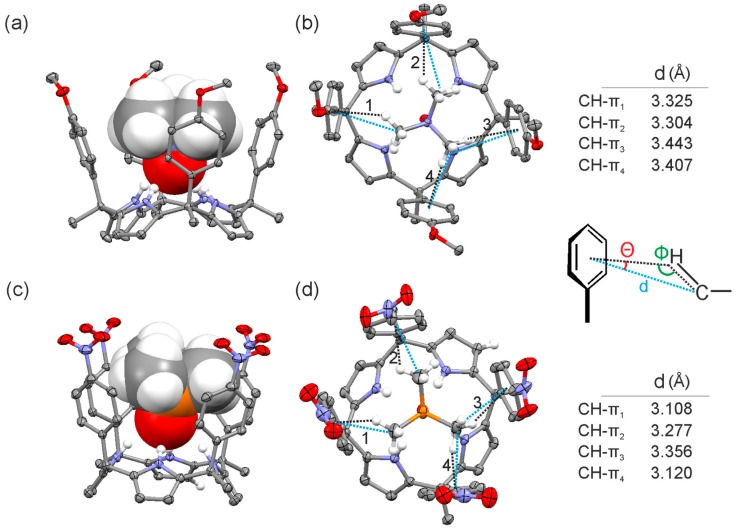
Side (**a**,**c**) and top (**b**,**d**) views of the X-ray structure of **2a**@**1e** and **2b**@**1f** complexes, respectively. Distances and angles related to the CH-π interactions are detailed.

Single crystals of the **2a**@**1e** complex suitable for X-ray diffraction grew from a millimolar acetonitrile solution by vapor diffusion of hexane. The X-ray structure showed the inclusion of trimethylamine *N*-oxide guest in the calix[4]pyrrole cavity establishing four hydrogen bonding interactions between the oxygen of the *N*-oxide group and the calix[4]pyrrole core (Figure 7a,b) and supported the binding geometry assigned to the complex in solution. Further analysis of the solid-state structure of the complex revealed that four CH-π interactions were possibly established between the methyl groups of the guest **2a** and the aromatic panels of the calix[4]pyrrole **1e** considering that the distance C_methyl_···Phenyl centroid lied within the range of values commonly used to describe these type of interactions in computational chemistry (Figure 7b). Based on this assumption, we assigned the energetic contribution of a single CH-π interaction a value of −1.1 (4.4/4) kcal·mol^−1^. This magnitude is in nice agreement with the typical ones reported in literature [4,5,15,30].

The titration of receptors **1e**–**f** in CD_3_CN solution with *P*-oxide **2b** showed a fast exchange kinetic behavior. The titration data were fitted to a theoretical 1:1 binding model using the HypNMR software [25]. The calculated association constant values were slightly higher than the ones calculated with the “two-wall” counterparts. The ΔΔ*G* values calculated for the CH-π interactions of the *P*-oxide **2b** using “four-wall” receptors did not double those determined using the “two-wall” system but showed a significant increase of 0.1–0.7 kcal·mol^−1^ that is in line with the formation of a more loose inclusion complex compared to that of **2a**.

X-ray diffraction analysis of single crystals of the **2b**@**1f** complex grown from acetonitrile solution by slow vapor diffusion of isobutanol revealed partial occupancy of the calix[4]pyrrole cavity with CH_3_CN and **2b** in a 80:20 molar ratio. The solid-state structure of the complex **2b**@**1f** (Figure 7c,d) revealed an analogous binding geometry of **2b** compared to **2a** in the **2a**@**1e** complex. The trimethylphosphine *P*-oxide **2b** is included in the calix[4]pyrrole cavity establishing four hydrogen bond interactions between the PO group and the calix[4]pyrrole core. Surprisingly, the distances between the C_methyl_ and the phenyl centroids related to the CH-π interactions were similar to the ones measured in the **2a**@**1e** complex. This may suggest that the cavity size of the receptor adapts to the volume of the included guest.

The solid-state structures of the inclusion complexes demonstrated deeper inclusion of the *N*-oxide guest **2a** in the calix[4]pyrrole cavity compared to the *P*-oxide **2b**. The N-H···O distances measured in both crystal structures were 2.095 and 2.254 Å, respectively. Most likely, the stronger hydrogen bonding interactions of **2a** with the pyrrole NHs yielded a complex with a conformationally rigid calix[4]pyrrole scaffold, which produced strong and fixed CH-π interactions between the methyl groups of the guest **2a** and the aromatic walls. Conversely, the weaker hydrogen bonding interactions of **2b** with the calix[4]pyrrole core of the aryl extended receptor were associated with fluxional aromatic walls that reduced the strength of the CH-π interactions with **2b**. The higher ordered and organized complexes produced by the “four-wall” receptors including guest **2a** could also explain the observed stronger CH-π interactions.

## 3. Experimental Section

The syntheses of the “two-wall” calix[4]pyrrole receptors used for this work (**1b**–**1d** from Figure 1) have been previously described in the literature by our group and others [16,31,32].

“Four-wall” calix[4]pyrrole receptors were prepared using described literature procedures [17,18,33]. NMR titrations were carried out on a Bruker 400 MHz spectrometer in CD_3_CN. Association constants (lower than 10^4^ M^−1^) were determined from the fit of the chemical shift changes of the different host protons in the NMR spectra with different amounts of guest using HypNMR software.

ITC data were obtained on a VP-ITC microcalorimeter, MicroCal, LLC (Northampton, MA, USA). The calorimetric titrations were performed by the injection of 3 μL aliquots of a solution of trimethylamine *N*-oxide **2a**, approximately seven times more concentrated than calix[4]pyrrole host solution (**1e** and **1f**) placed in the cell (T = 293 K). After the reference titration was subtracted, the association constants and the thermodynamic parameters were obtained from the fit of the revised titration data to a theoretical titration curve of a 1:1 binding model. The values were calculated using Origin 7 software package which uses least-squares minimization to obtain globally optimized parameters as described in Wiseman *et al.* [34].

CCDC 1417349-1417350 contain the supplementary crystallographic data for this paper. These data can be obtained free of charge via https://summary.ccdc.cam.ac.uk/structure-summary-form (or from the CCDC, 12 Union Road, Cambridge CB2 1EZ, UK; Fax: +44-122-333-6033; E-Mail: deposit@ccdc.cam.ac.uk).

## 4. Conclusions

In summary, we have quantified the contribution of CH-π interactions in the thermodynamic stability of “two-wall” and “four-wall” calix[4]pyrrole inclusion complexes with trimethylamine *N*-oxide **2a** and trimethylphosphine *P*-oxide **2b**. We demonstrated that the magnitude of the CH-π interactions established between the guests and the aromatic panels of “two/four-wall” calix[4]pyrrole receptors did not depend on the electronic nature of the phenyl walls. This observation indicated that electrostatic factors are not relevant in these CH-π interactions, which should be mainly dominated by dispersion forces. Most likely, the reduced hydrogen-bond donor properties (low alpha values) of the methyl protons of the guests render the studied contacts as “typical” CH-π interactions that are known to be mainly dominated by London dispersion forces.

We also noticed that the contribution of CH-π interactions to the total free energy of binding of the inclusion complexes is larger with trimethylamine *N*-oxide **2a** than with *P*-oxide **2b**. We attributed this significant effect to differences in the hydrogen bonding strengths of both guests with the calix[4]pyrrole core of the receptors, which is translated in subtle differences on the arrangements of the methyl groups with respect to the aromatic walls. In addition, we hypothesize that the stronger binding exhibited by the *N*-oxide **2a** with the calix[4]pyrrole receptors produce conformationally more rigid complexes reinforcing the magnitudes of the CH-π interactions between the methyl groups of the guest **2a** and the aromatic walls. Based on the number of CH-π interactions observed in the solid-state structure of the “four-wall” inclusion complex **2a**@**1e**, we estimated the energetic contribution of a single CH-π interaction (Δ*G* ~ 1.1 kcal·mol^−1^). Our estimate was in complete agreement with other values previously described in the literature for “typical” CH-π interactions.

## Supplementary Materials

^1^H-NMR titration experiments, ITC titration experiments, fit of the titration data, and representation of the ESP of the aromatic walls of calix[4]pyrroles **1b**–**1d**. Supplementary materials can be accessed at: http://www.mdpi.com/1420-3049/20/09/16672/s1.
