# Supplementary material for: Quantification of CH-π Interactions Using Calix[4]pyrrole Receptors as Model Systems
Source: Molecules. 2015 Sep 14;20(9):16672–86. doi: 10.3390/molecules200916672 (PMC6332063; doi:10.3390/molecules200916672)
Supplement: Supplementary file 1 [file molecules-20-16672-s001.pdf]

Supporting Information

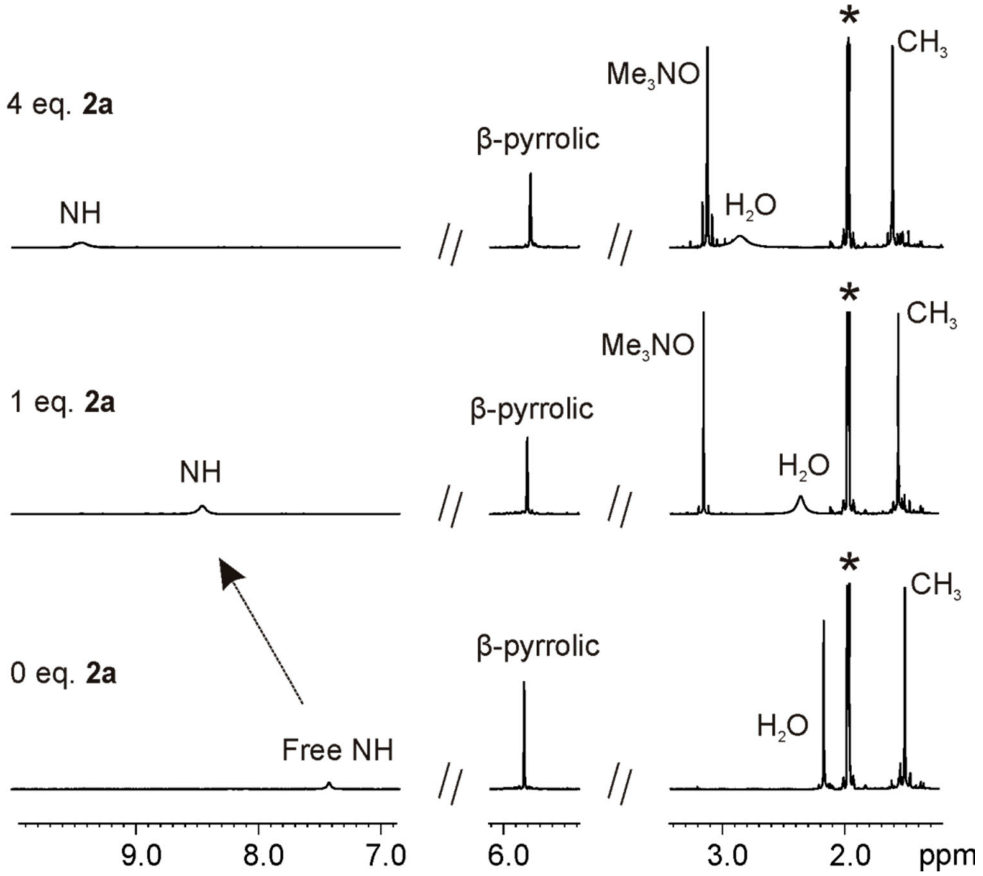

Figure S1. Changes in the ^{1}H-NMR spectra during the titration of **1a** with **2a** in CD_3CN. * signal for the proton in partially deuterated solvent CHD_2CN.

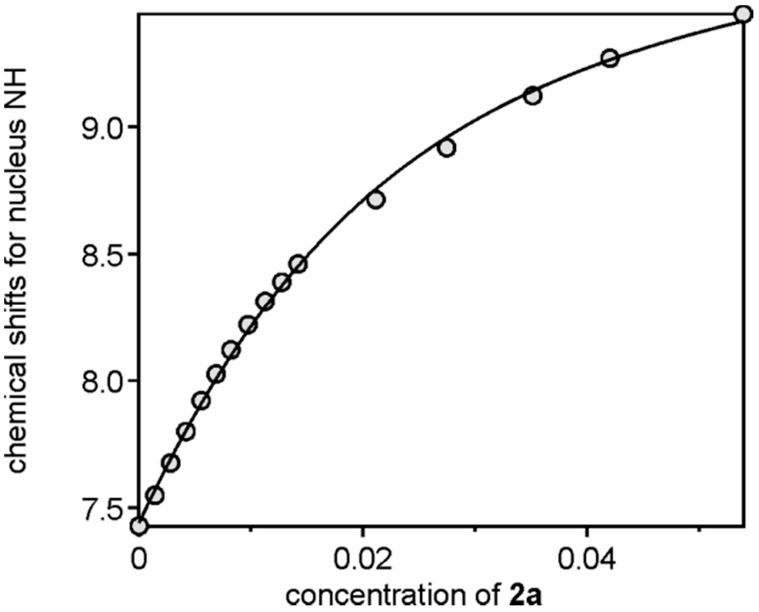

Figure S2. Fit of the chemical shift changes, experienced by NH protons of **1a** during the titration with **2a**, using a 1:1 binding model (line) implemented in the HypNMR2008 software.

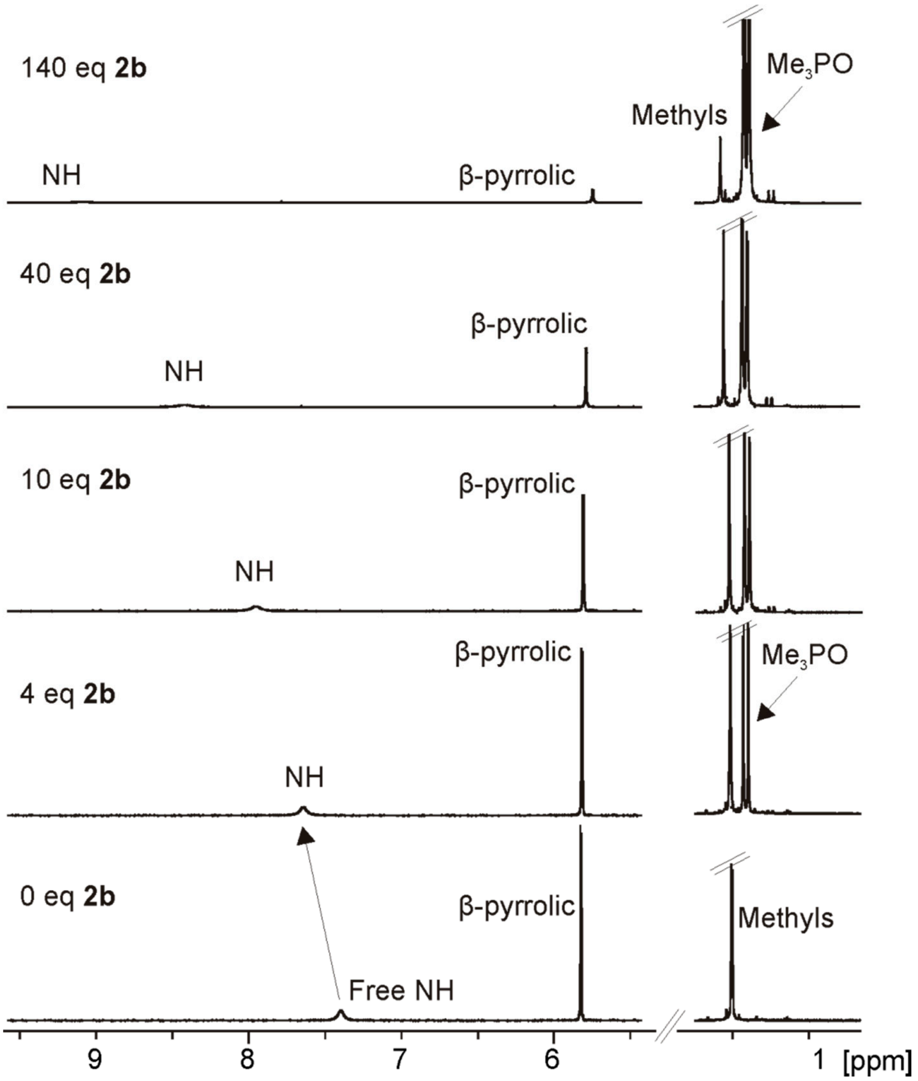

Figure S3. Changes in the ^{1}H-NMR spectra during the titration of **1a** with **2b** in CD_3CN.

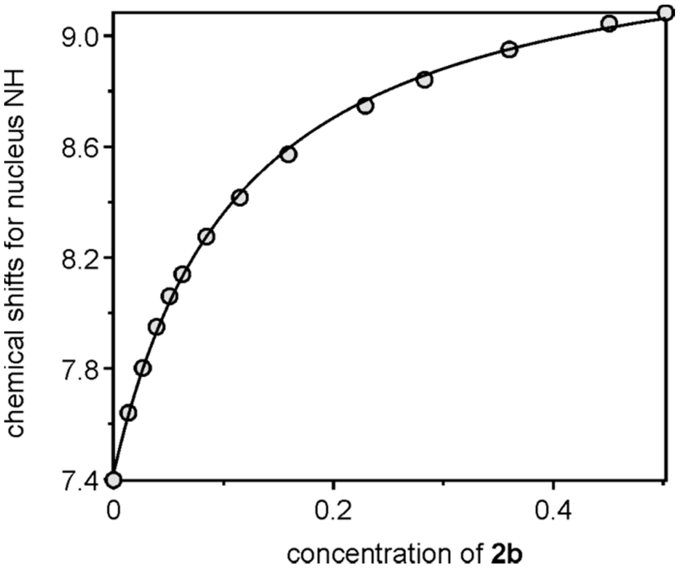

Figure S4. Fit of the chemical shift changes, experienced by NH protons of 1a during the titration with **2b**, using a 1:1 binding model (line) implemented in the HypNMR2008 software.

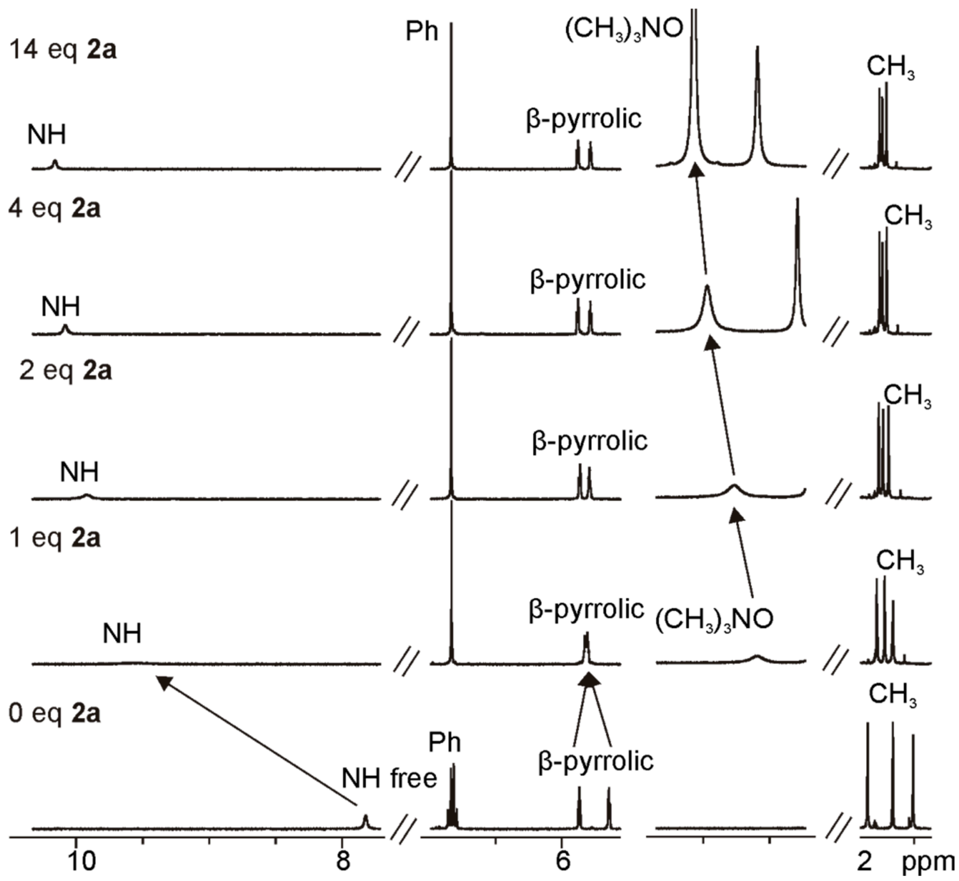

Figure S5. Changes in the ^{1}H-NMR spectra during the titration of **1b** with **2a** in CD3CN.

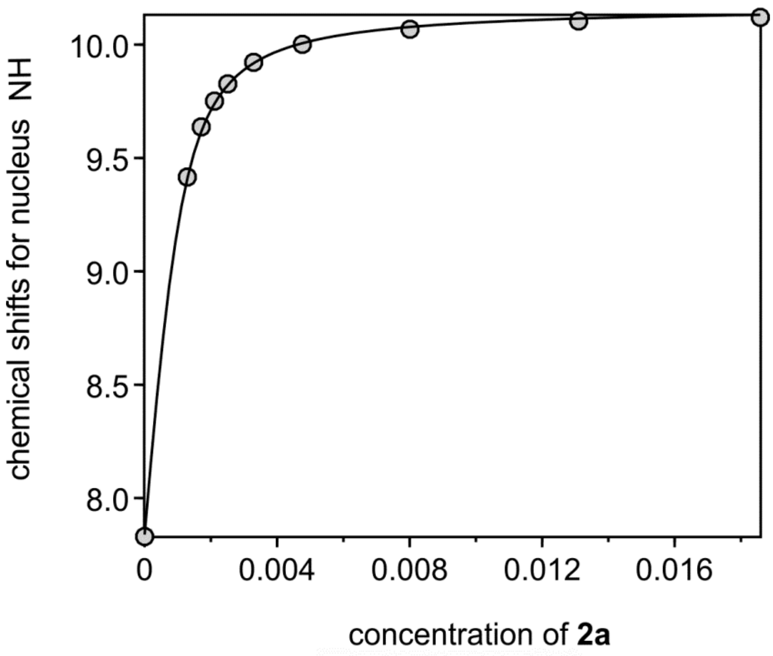

Figure S6. Fit of the chemical shift changes, experienced by NH protons of **1b** during the titration with **2a**, using a 1:1 binding model (line) implemented in the HypNMR2008 software.

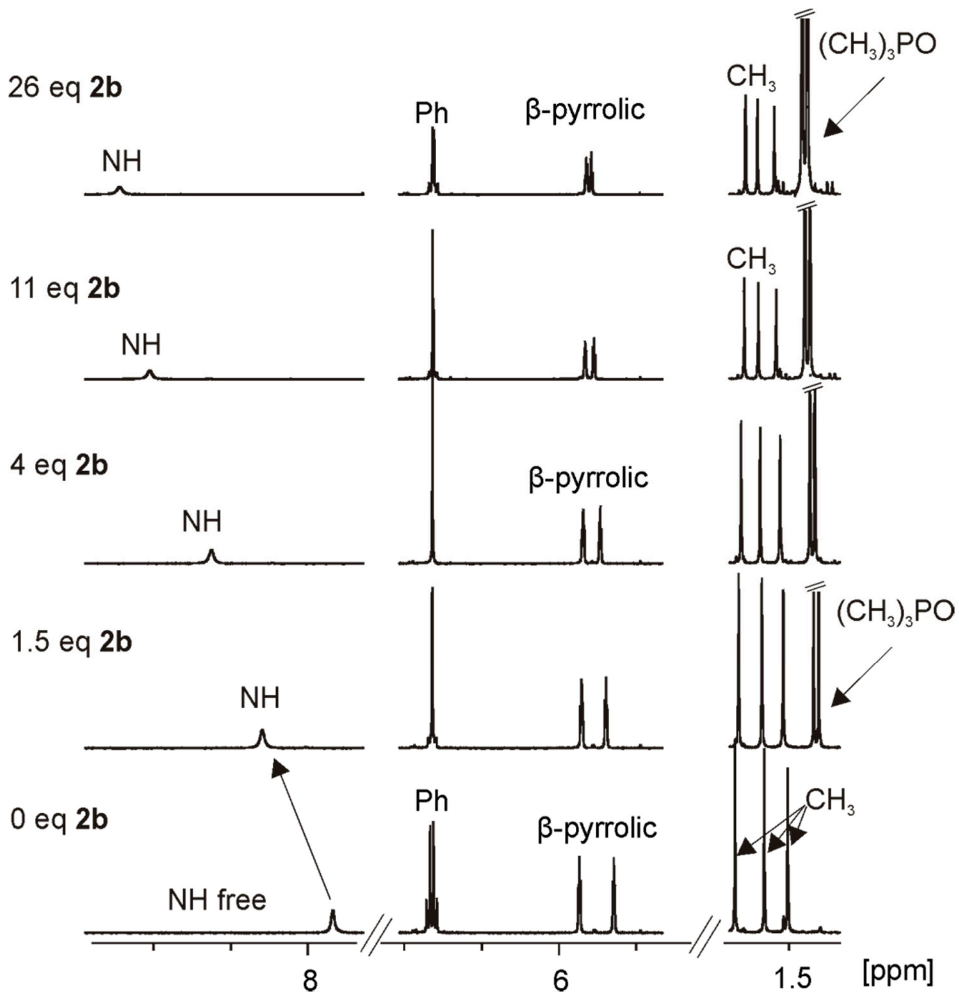

Figure S7. Changes in the ^{1}H-NMR spectra during the titration of **1b** with **2b** in CD_3CN.

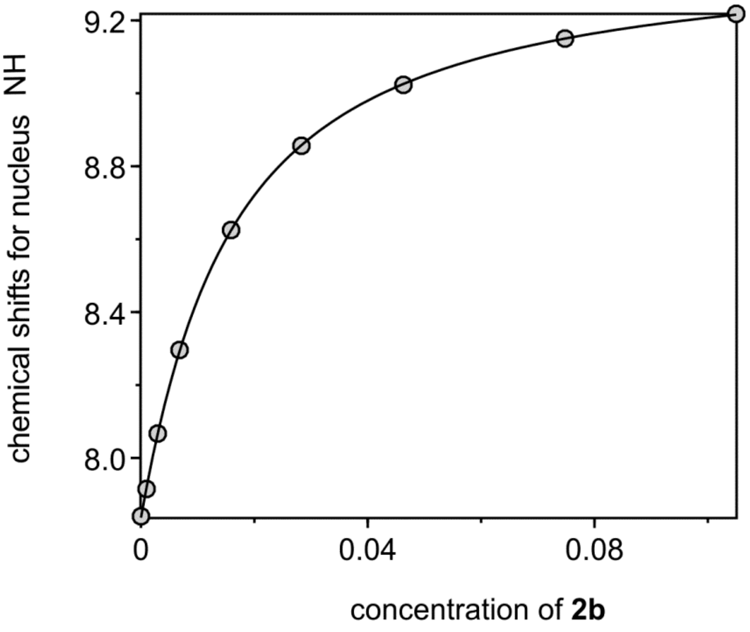

Figure S8. Fit of the chemical shift changes, experienced by NH protons of **1b** during the titration with **2b**, using a 1:1 binding model (line) implemented in the HypNMR2008 software.

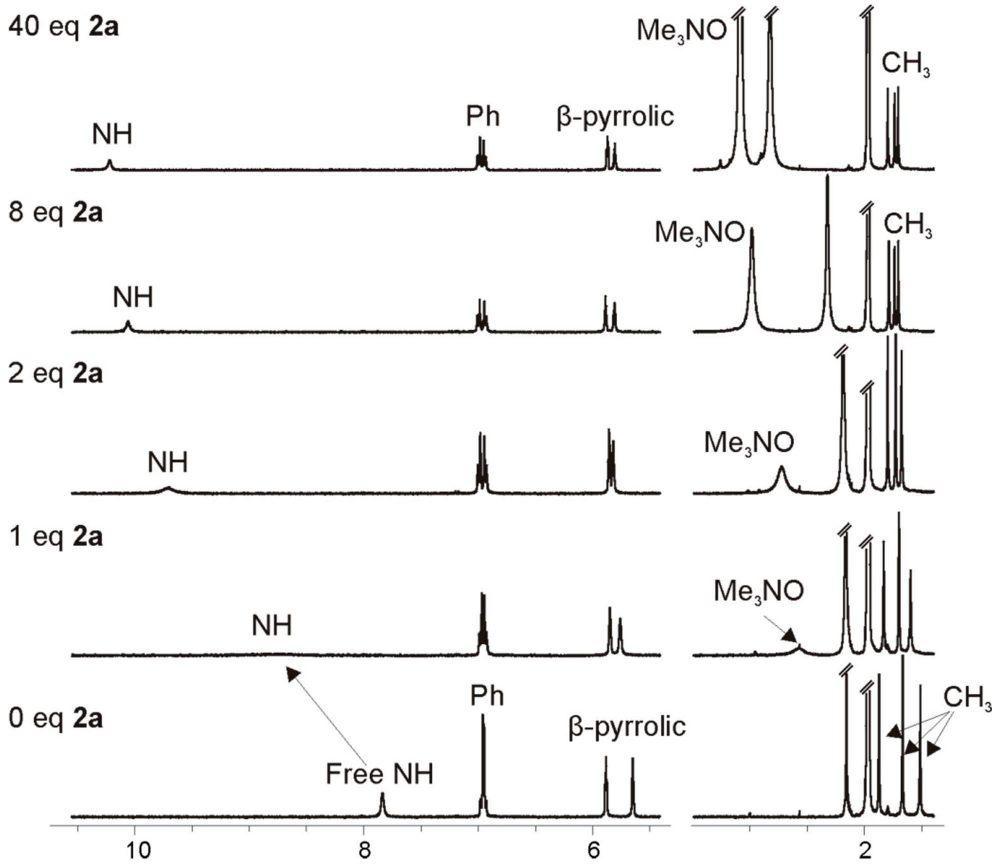

Figure S9. Changes in the ^{1}H-NMR during the titration of **1c** with **2a** in CD_3CN.

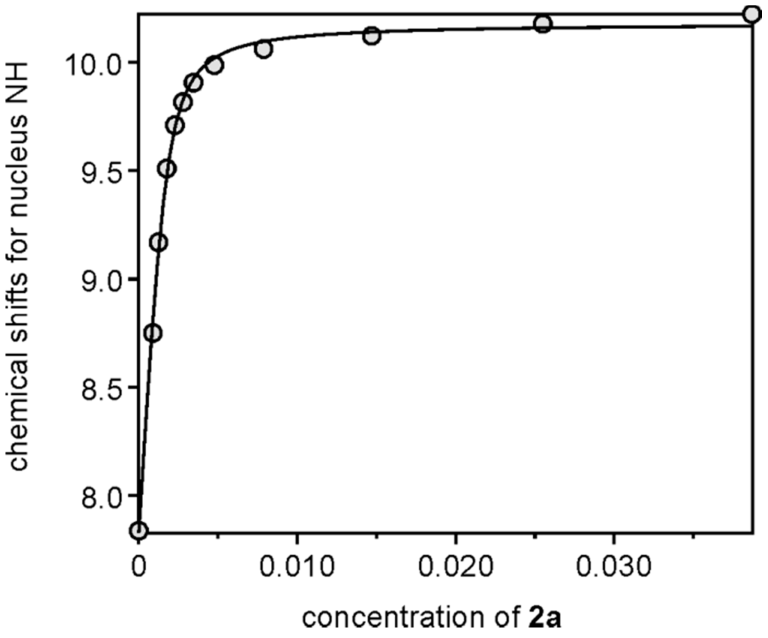

Figure S10. Fit of the chemical shift changes, experienced by NH protons of **1c** during the titration with **2a**, using a 1:1 binding model (line) implemented in the HypNMR2008 software.

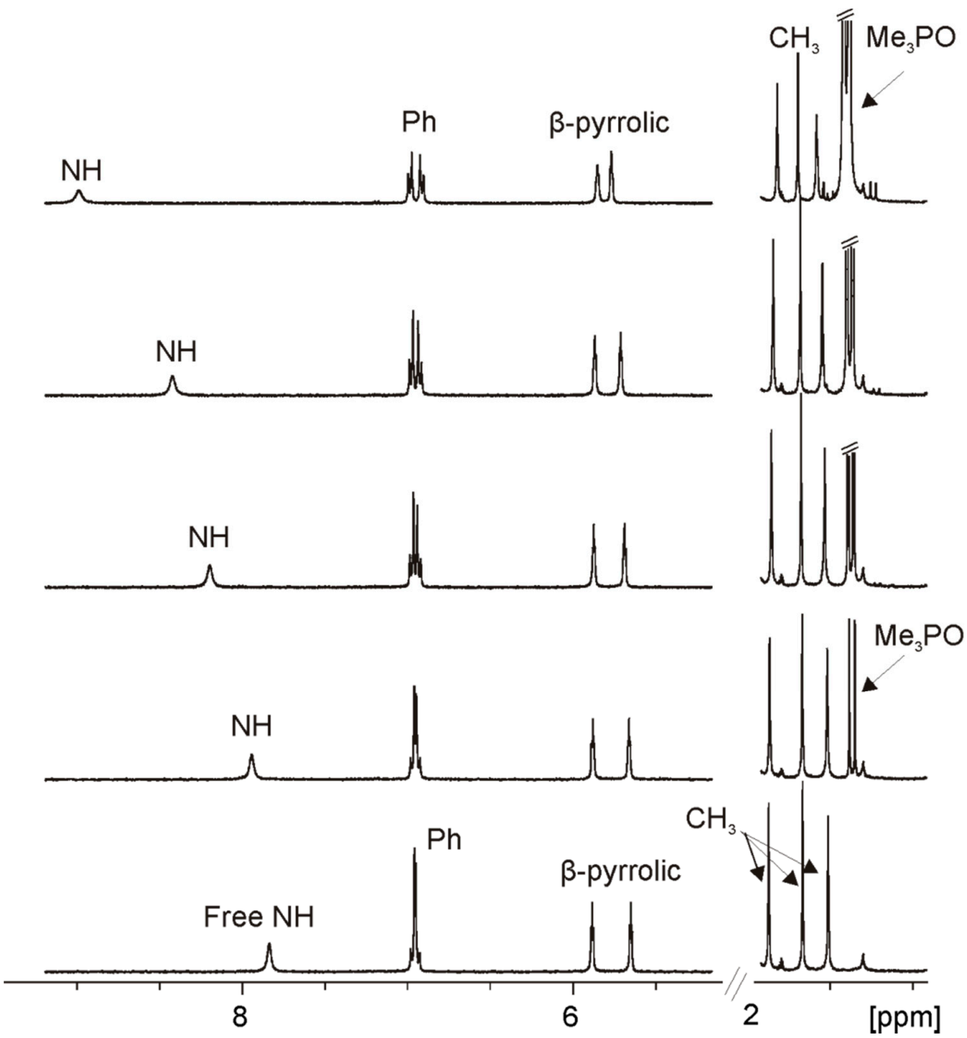

Figure S11. Changes in the ^{1}H-NMR during the titration of **1c** with **2b** in CD_3CN.

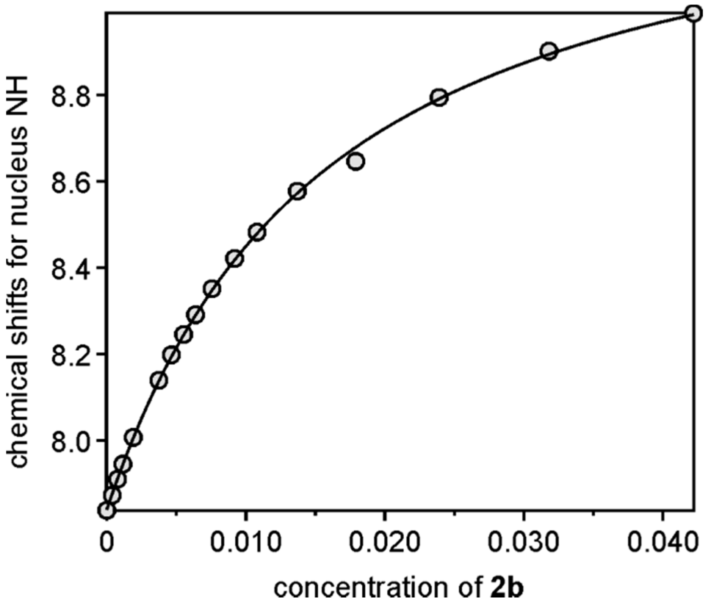

Figure S12. Fit of the chemical shift changes, experienced by NH protons of **1c** during the titration with **2b**, using a 1:1 binding model (line) implemented in the HypNMR2008 software.

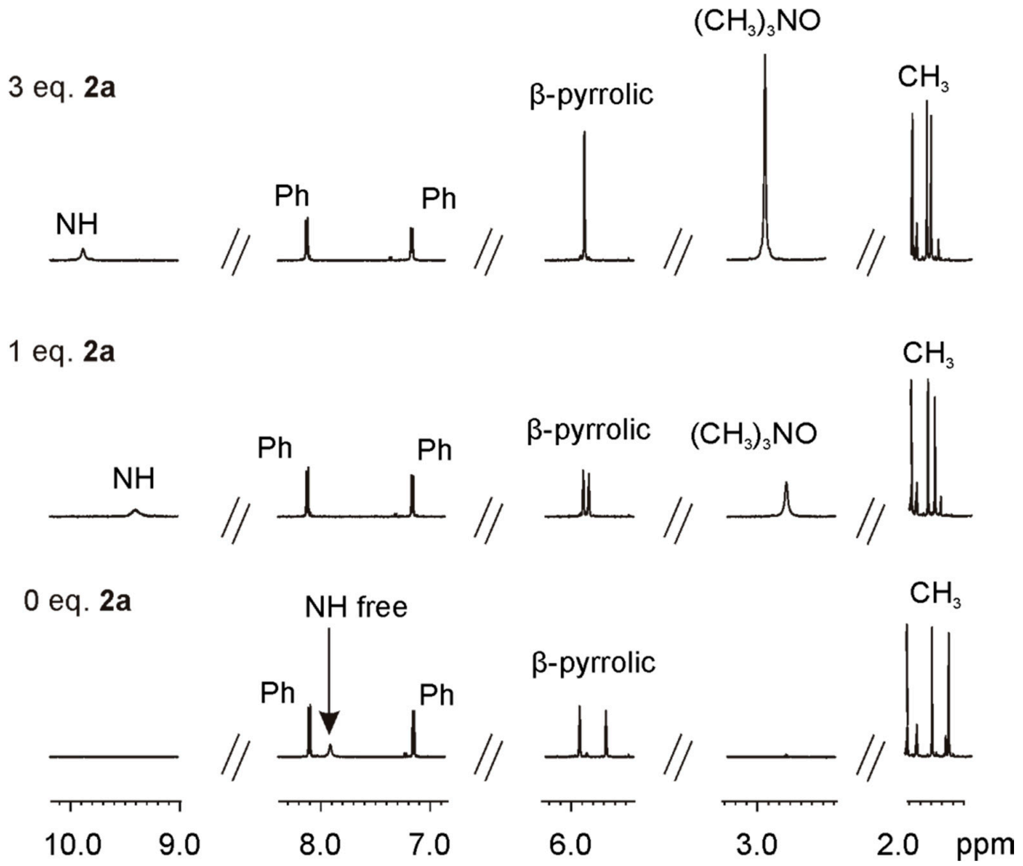

Figure S13. Changes in the ^{1}H-NMR spectra during the titration of **1d** with **2a** in CD_3CN.

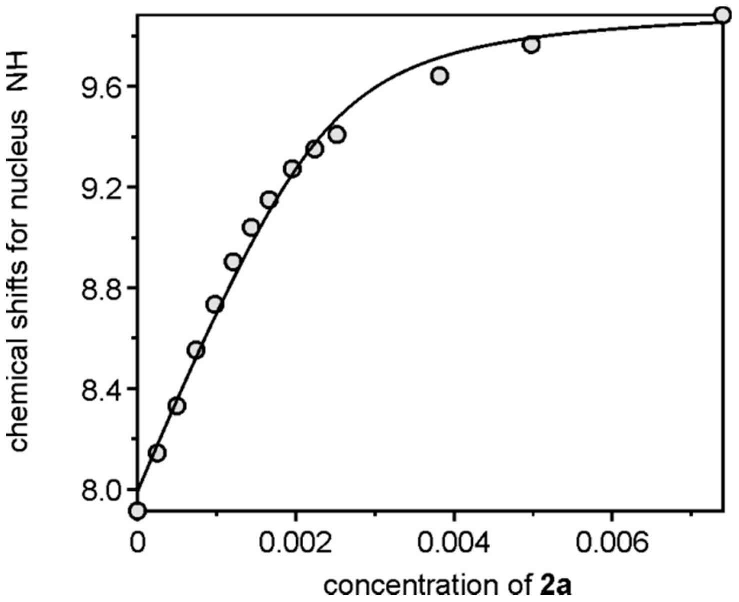

Figure S14. Fit of the chemical shift changes, experienced by NH protons of **1d** during the titration with **2a**, using a 1:1 binding model (line) implemented in the HypNMR2008 software.

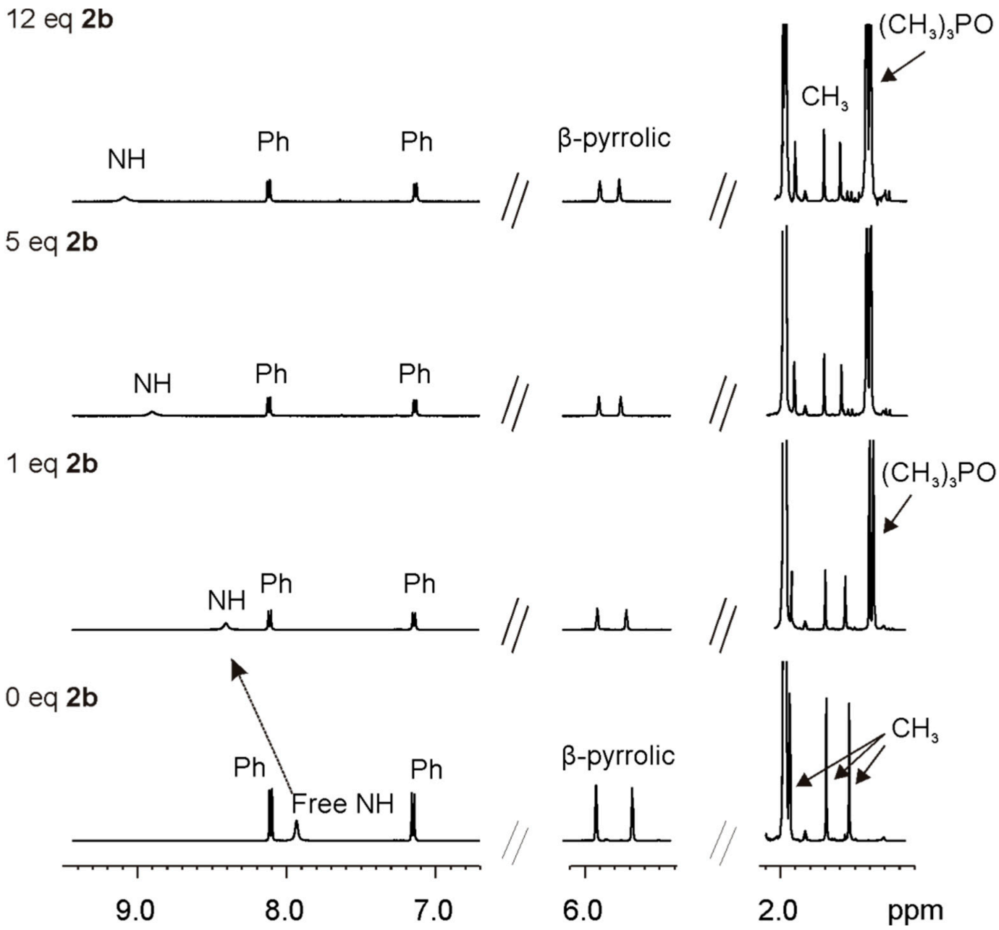

Figure S15. Changes in the ^{1}H-NMR spectra during the titration of **1d** with **2b** in CD_3CN.

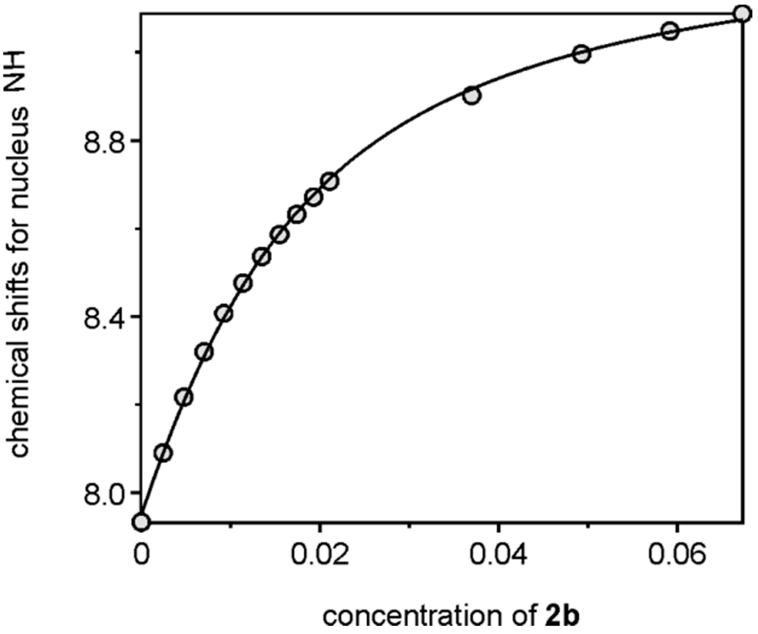

Figure S16. Fit of the chemical shift changes, experienced by NH protons of **1d** during the titration with **2b**, using a 1:1 binding model (line) implemented in the HypNMR2008 software.

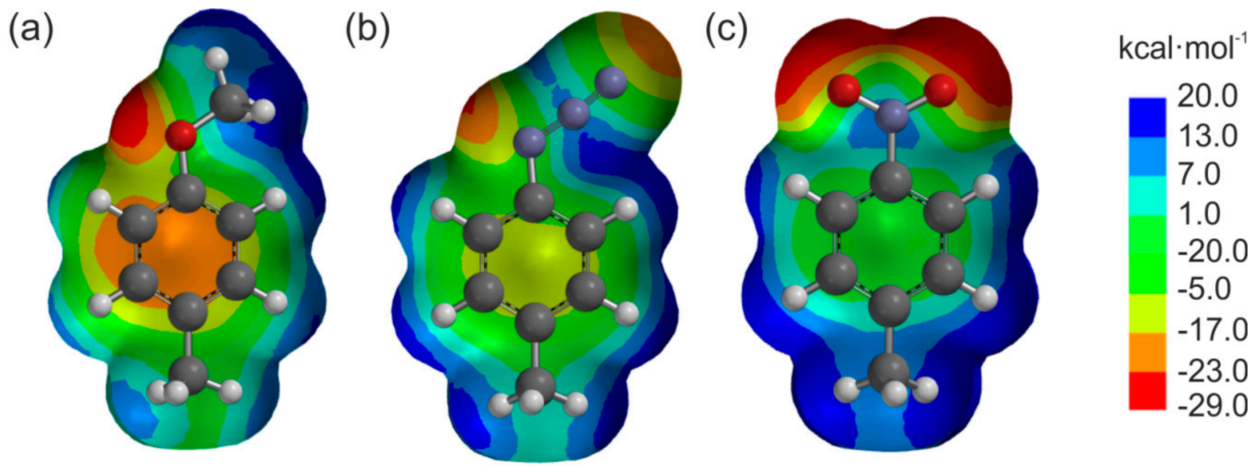

Figure S17. Representation of the electrostatic potential on the Van der Waals surface corresponding to the aromatic walls of **1b** (**a**) **1c** (**b**) and **1d** (**c**). The surface was calculated by using B3LYP 6-31G* in vacuum.

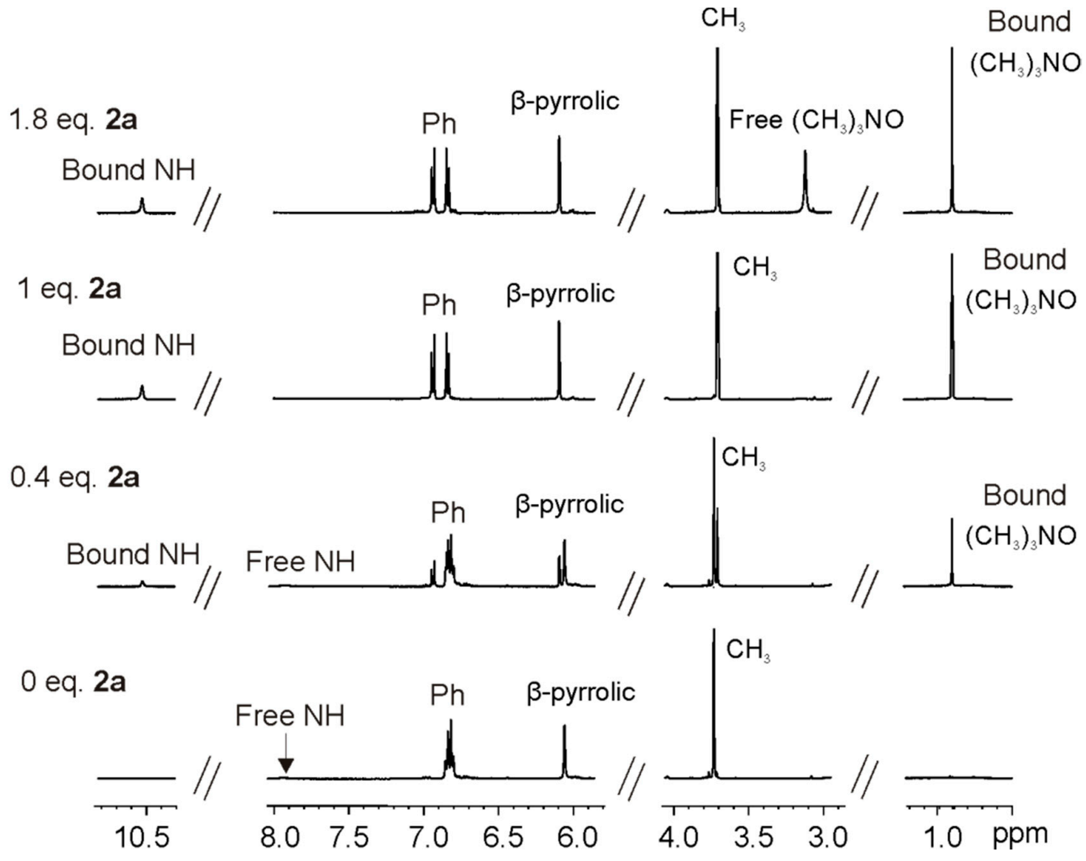

Figure S18. Changes in the ^{1}H-NMR spectra during the titration of **1e** with **2a** in CD_3CN.

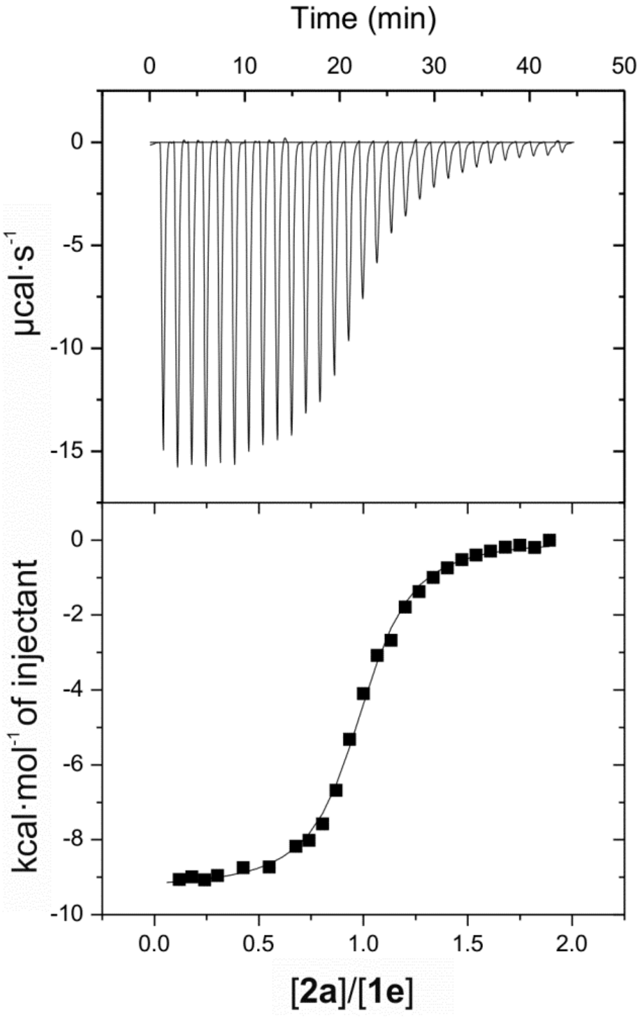

Figure S19. ITC titration experiment of the formation of the **2a** ⊂ **1e** complex. **Top**: rawdata; **Bottom**: normalized integration data of the evolved heat per injection in terms of kcal·mol^{-1} of injectant (**2a**) plotted against the molar ratio **2a**–**1e**. To determine the values of the thermodynamic variables the ITC data have been fitted to a 1:1 binding model (black line).

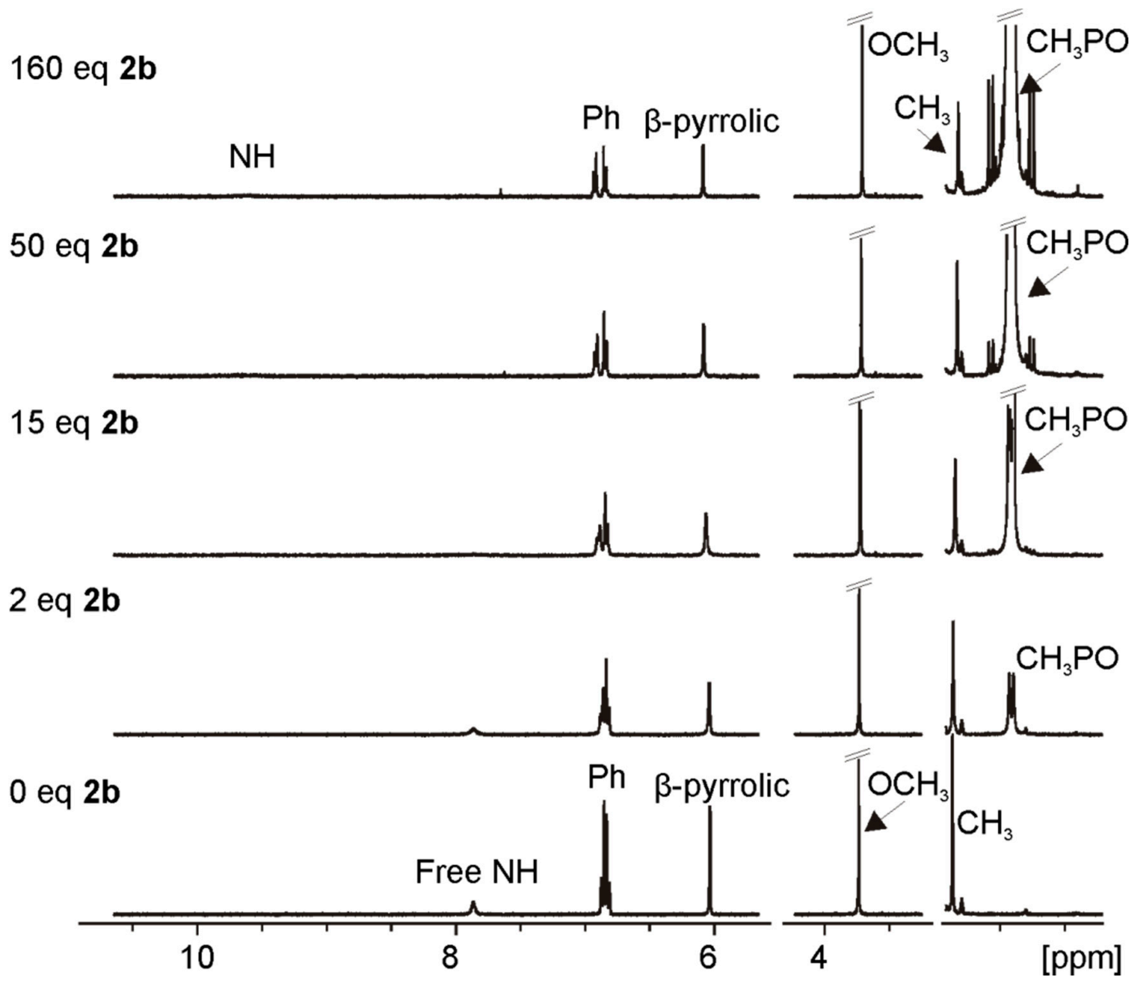

Figure S20. Changes in the ^{1}H-NMR spectra during the titration of **1e** with **2b** in CD3CN.

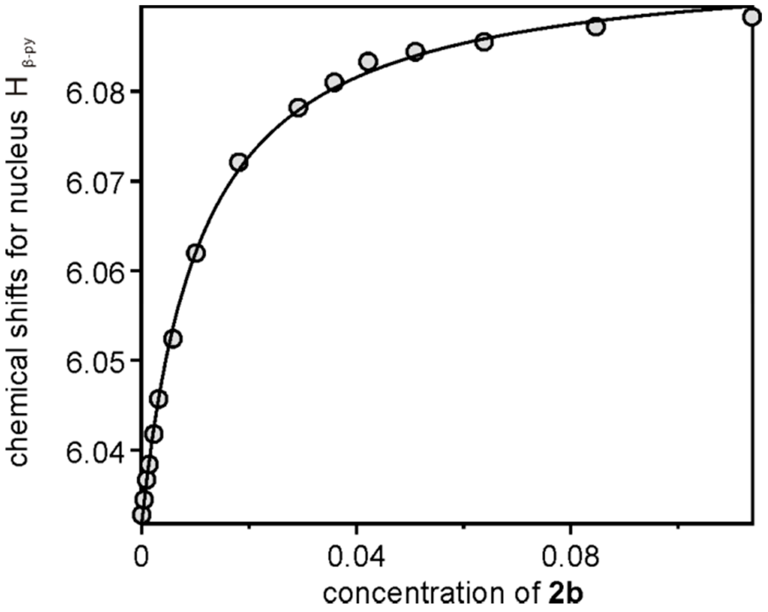

Figure S21. Fit of the chemical shift changes, experienced by NH protons of 1e during the titration with **2b**, using a 1:1 binding model (line) implemented in the HypNMR2008 software.

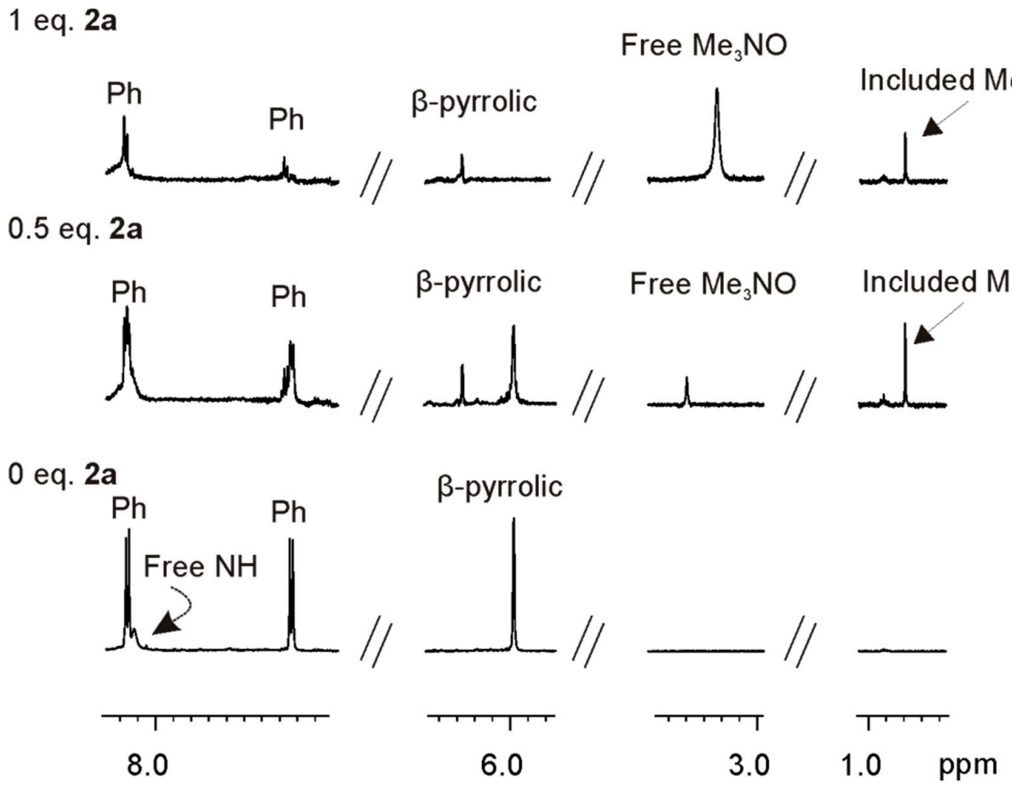

Figure S22. Changes in the ^{1}H-NMR spectra during the titration of **1f** with **2a** in CD_3CN.

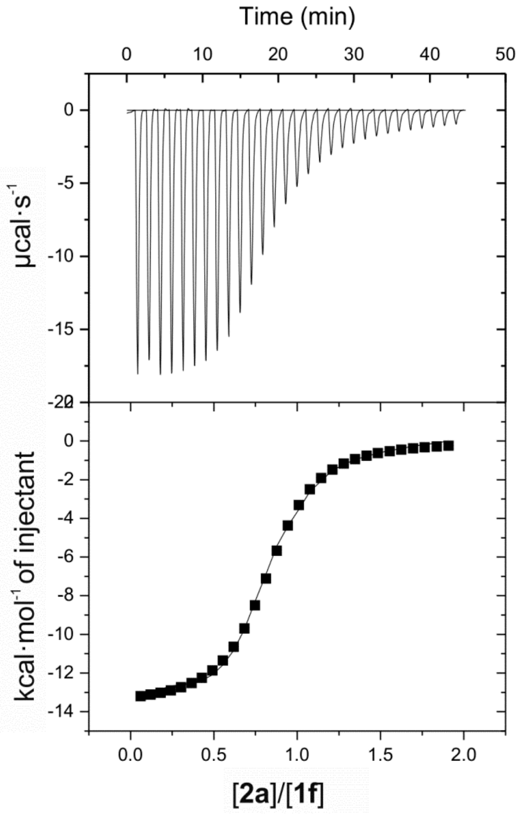

Figure S23. ITC titration experiment of the formation of the **2a** ⊂ **1f** complex. Top: rawdata. Bottom: normalized integration data of the evolved heat per injection in terms of kcal·mol^{-1} of injectant (**2a**) plotted against the molar ratio **2a**–**1f**. To determine the values of the thermodynamic variables the ITC data have been fitted to a 1:1 binding model (black line).

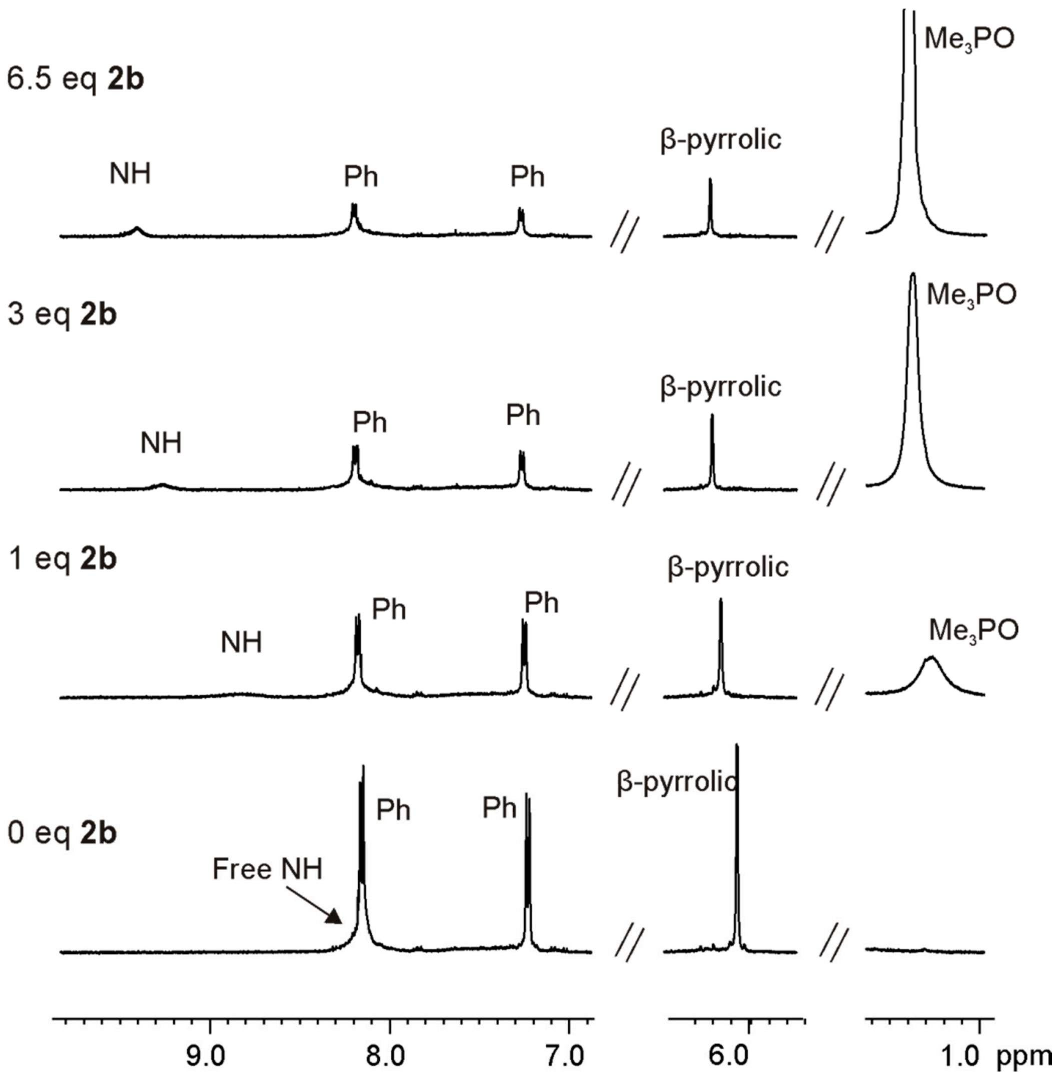

Figure S24. Changes in the ^{1}H-NMR spectra during the titration of **1f** with **2b** in CD_3CN.

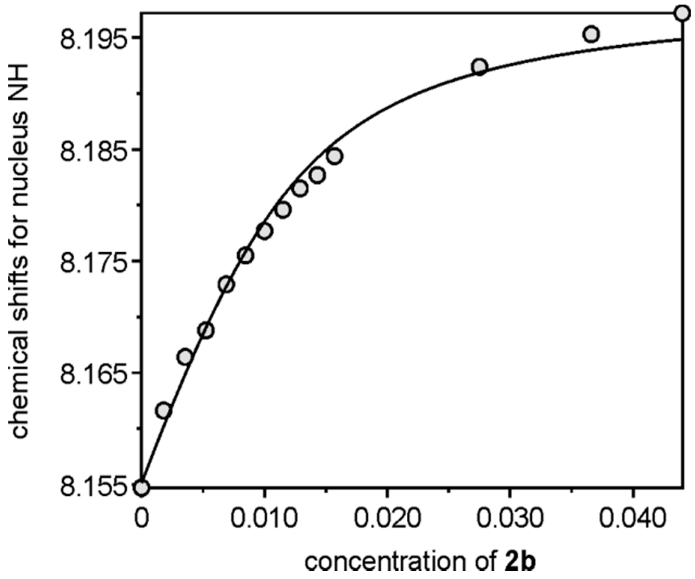

Figure S25. Fit of the chemical shift changes, experienced by NH protons of **1f** during the titration with **2b**, using a 1:1 binding model (line) implemented in the HypNMR2008 software.
